# Prenatal evaluation of fetal atrioventricular valves by real-time 4D volume imaging with electronic matrix probe

**DOI:** 10.1186/s12947-021-00240-7

**Published:** 2021-01-28

**Authors:** Huiyu Tang, Wei Sun, Xue Sun, Yu Wang, Yu Qi, Dong Wang, Ying Zhang

**Affiliations:** Department of Ultrasound, Shengjing Hospital of China Medical University, No. 36 Sanhao Street, Heping District Shenyang, 110004 People’s Republic of China

**Keywords:** Electronic matrix probe, RT 4D, Atrioventricular valves, Prenatal diagnosis, Fetal heart

## Abstract

**Background:**

The purpose of this study is to evaluate the feasibility using real-time four-dimensional (RT 4D) volume imaging with electronic matrix probe to observe the morphology of atrioventricular valves in normal and abnormal fetuses, measure the area and circumference of atrioventricular valves in normal fetuses and analyze the correlation with gestational age.

**Methods:**

RT 4D volume imaging with electronic matrix probe was used to collect cardiac volume data of 162 normal fetuses with the gestational age from 22 to 32 weeks and 19 fetuses with atrioventricular valves abnormalities were also enrolled. All the volume data were analyzed and processed in real-time. The morphology of mitral and tricuspid valves was observed in surface mode. The area and circumference of valves were measured in a 4D render view at the end of diastole and analyzed the correlation with gestational age.

**Results:**

In 148 of 162 fetuses (91%), the 4D rendered image could be successfully obtained, which clearly showed the morphology of the atrioventricular valves. The area and circumference of mitral and tricuspid valves were positively correlated with gestational age (*P* < 0.01). Furthermore, 4D rendered images were successfully obtained in 17 of 19 fetuses (89%) with atrioventricular valves abnormalities.

**Conclusions:**

The reference range of the area and circumference of atrioventricular valves in normal fetuses at different gestational weeks could be determined by using the RT 4D volume imaging with electronic matrix probe, which can provide certain diagnostic information for the clinic. The RT 4D images could display the valves morphology vividly in both normal and abnormal fetuses, including some subtle lesions which are not identified by traditional two-dimensional (2D) echocardiography. It is feasible to use the RT 4D volume imaging with electronic matrix probe to perform the prenatal evaluation in the fetal atrioventricular valves.

**Supplementary Information:**

The online version contains supplementary material available at 10.1186/s12947-021-00240-7.

## Background

Congenital heart disease (CHD) is one of the most common congenital anatomical malformations, which accounting for about 0.6% of all live births [1, 2]. Atrioventricular valves abnormalities are common types of heart defect [3]. As the important anatomical structure connecting atrium and ventricle, atrioventricular valves have important hemodynamic significance. The abnormal valves will occur stenosis, reflux, and even cause secondary changes such as volume load in severe cases.

For more than 10 y, spatial temporal correlation imaging (STIC) has made significant contributions in the field of fetal heart [4–9]. The STIC technology has added some advantages for fetal cardiac examination: visualizing structures which are not identified by 2D echocardiography; ability to get depth perception in rendered images; obtained the standard 2D planes easily from 4D volumes; improved patient counseling; and offline analysis of datasets for expert review and telemedicine [6, 10]. Recent years, 4D ultrasound has achieved further development, especially the advent of the electronic matrix probe, which has strengthened the diagnostic ability of fetal heart diseases [11, 12]. The most attractive is the ability to obtain and analyze fetal heart volume data in real-time, which makes prenatal examination enter a real real-time field and is expected to be widely used in fetal echocardiography. As a new technology with fast acquisition speed and high image resolution, the RT 4D volume imaging can evaluate the anatomical structure and functional status of fetal heart in real-time from multiple angles by real-time analyzing and processing the collected volume data. In this study, we used the electronic matrix probe RT 4D volume imaging to dynamically observe the morphology of the normal fetal atrioventricular valves and measure the area and circumference of the normal fetal atrioventricular valves to evaluate its correlation with the gestational weeks. Some abnormal atrioventricular valves were also visualized by RT 4D mode.

## Methods

### Study population

Between January 2018 to December 2019, 162 normal fetuses and 19 abnormal fetuses who underwent fetal heart examination in Shengjing Hospital of China Medical University signed the informed consent and participated in this study involving the utilization of the RT 4D volume imaging in fetal echocardiography. In normal fetuses, the gestational age at fetal diagnosis ranged from 22 to 32 w (mean, 26.6 ± 2.9) and the maternal age ranged from 21 to 39 years (mean, 30.2 ± 4.1). In fetuses with atrioventricular valves abnormalities, the gestational age at fetal diagnosis ranged from 20 to 29 w (mean, 24.2 ± 2.5) and the maternal age ranged from 21 to 41 years (mean, 30.4 ± 6.1). All the fetuses were singletons. The quality of the 2D images during the fetal heart examination was good. The examination, data collection, real-time analysis and processing were all performed by one sonographer. Fetal heart RT 4D volume datasets were obtained in most patients with suspected cardiac anomalies. Autopsy findings from terminated pregnancies were compared to fetal echocardiograms.

### Conventional fetal echocardiography

All fetuses were examined using a 4D ultrasound system (Voluson E10, GE Healthcare, Kretztechnik, Zipf, Austria), equipped with an electronic matrix probe (eM6C) with RT 4D volume imaging. Conventional 2D scanning was performed on each fetus to obtain the four-chamber view (4CV), left and right outflow tract views, three-vessel view and three-vessel-trachea view for routine analysis.

### RT 4D volume imaging

#### Volume acquisition

RT 4D volume imaging with electronic matrix probe was performed in all normal and abnormal fetuses. The frame rate was set to high level and image acquisition was started when a clear 4CV of the fetuses was obtained. The volumes contained the anatomical structures between the gastric vacuole and fetal upper mediastinum. The sweep angle ranged from 25 to 40°. The volumes were immediately reconstructed and analyzed in real-time (4D Viewer, version 14.0; GE Medical Systems).

#### 4D volume real-time analysis

**Fig. 1:**
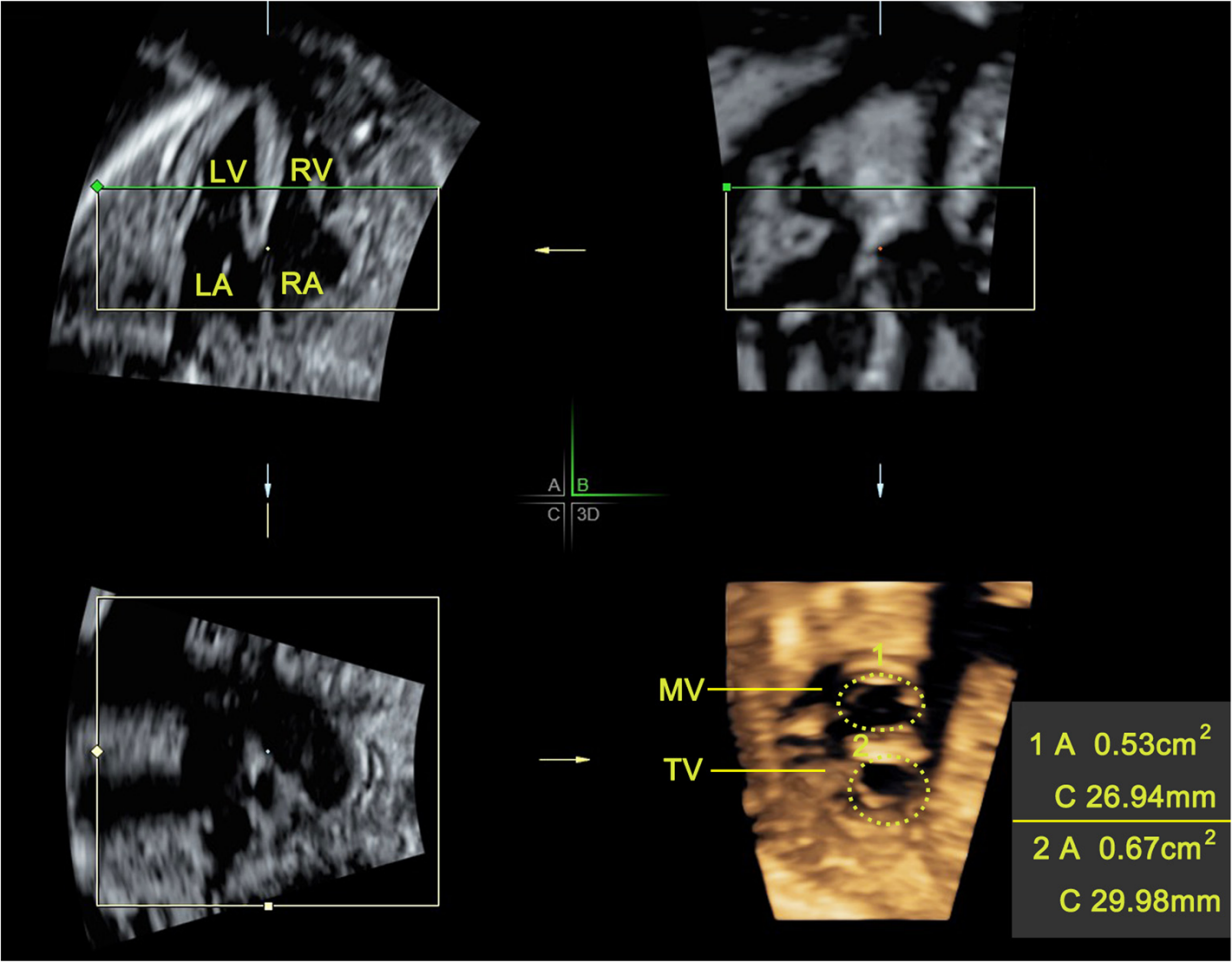
The 4D rendering images of the atrioventricular valves (mitral and tricuspid) are displayed at the D (3D) plane. Measure the area and circumference of the atrioventricular valves using RT 4D volume imaging with electronic matrix probe at the end of diastole. LA, left atrium; LV, left ventricle; MV, mitral valve; RA, right atrium; RV, right ventricle; TV, tricuspid valve

All volumes were analyzed and processed by one sonographer in real-time. All volume datasets were displayed in the multiplanar modality. Three orthogonal planes (transverse panel A, sagittal panel B and coronal panel C) were presented simultaneously. Click “Render” to perform RT 4D volume reconstruction. Enable the surface mode and adjust the reference point of plane A to be placed at the midpoint of the mitral and tricuspid annulus. Adjusting the size of region of interest (ROI) to ensure the mitral and tricuspid valves were included. Appropriately adjust the X, Y and Z axes to make the mitral and tricuspid annuluses horizontal in plane A and B. Place the green line of ROI on the ventricular side and as close as possible to the mitral or tricuspid annulus. A RT 4D image of the atrioventricular valves were in panel D (3D). A combination of smooth surfaces and gradient light algorithms and real-time processing adjustments to improve image quality. The area and circumference of the mitral and tricuspid valves are measured at the end of diastole with manual tracing for the three times by one sonographer and taken the average value finally (Fig. 1).

### Statistical analysis

Data were expressed as means ± standard deviation (SD). Pearson correlation analysis was used to evaluate the correlation between the area and circumference of the mitral, tricuspid valves and the gestational age. *P* < 0.05 was statistically significant.

## Results

**Fig. 2:**
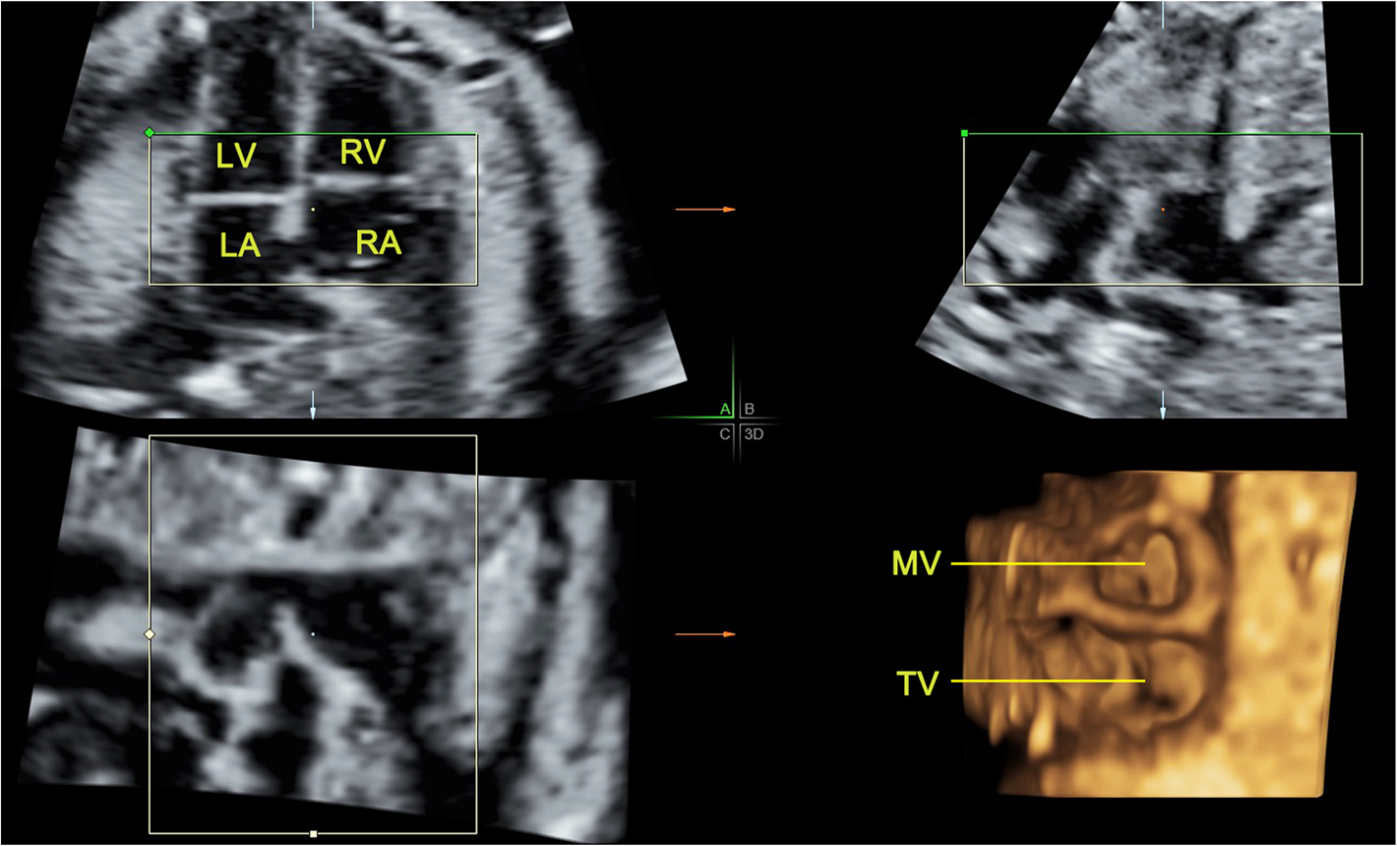
The 4D rendered image of the atrioventricular valves (mitral and tricuspid) retrieved by RT 4D volume imaging combine with surface mode in a normal fetus of 25 gestational weeks. The RT 4D image can clearly show the mitral valve with two leaflets and tricuspid valve with three leaflets during systole. There is no artifact on the B and C planes. LA, left atrium; LV, left ventricle; MV, mitral valve; RA, right atrium; RV, right ventricle; TV, tricuspid valve

**Fig. 3:**
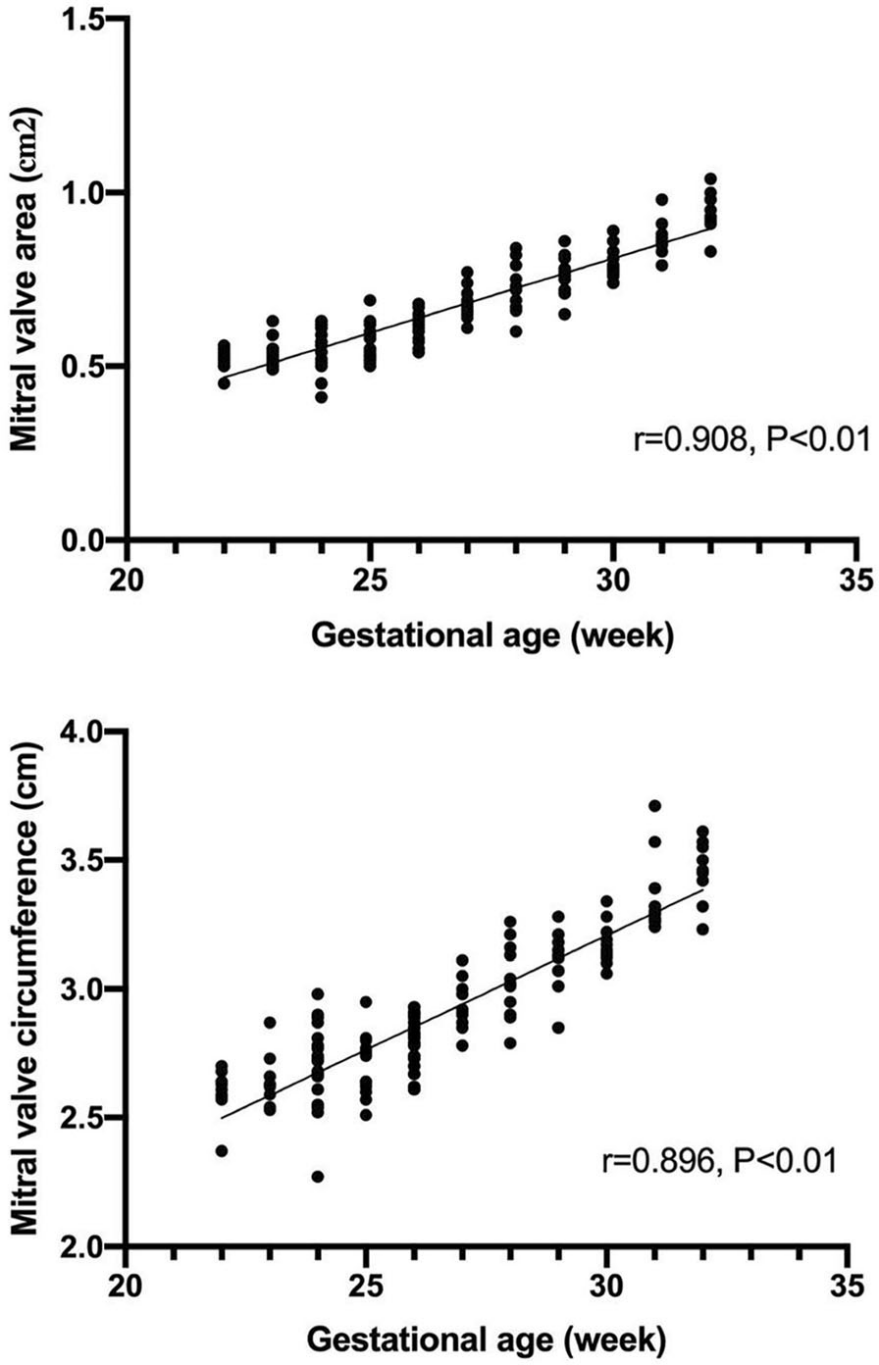
The scatter diagram for fetal mitral valve area and circumference according to gestational age

**Fig. 4:**
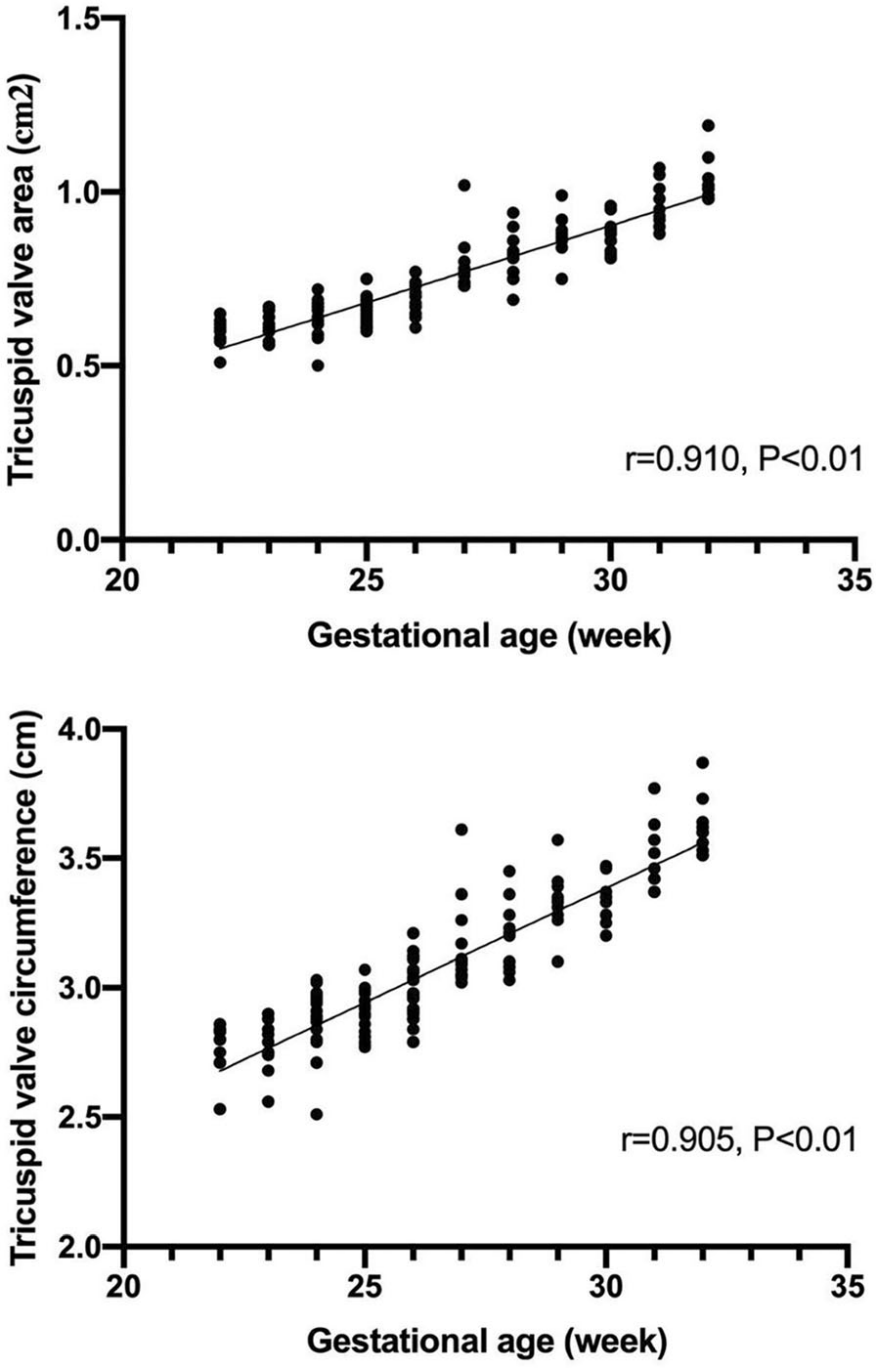
The scatter diagram for fetal tricuspid valve area and circumference according to gestational age

Of the 162 normal fetuses, 14 cases were failed to collect satisfactory volume data because of being affected by fetal position, amniotic fluid, spine and rib sound shadows. One hundred forty-eight cases were successfully obtained. When the 4D volumes were analyzed in real-time, the 4D rendered images of the mitral and tricuspid valves were obtained and presented simultaneously as the two valve annuluses were approximately at the same level. The 4D rendered images clearly showed the shape and the movement of the cardiac valves even the number of the leaflets in high-quality volume data (Fig. 2, Additional file 1). The area and circumference of mitral and tricuspid valves are positively correlated with gestational age (mitral valve area r = 0.908, *P* < 0.01; mitral valve circumference r = 0.896, *P* < 0.01(Fig. 3); tricuspid valve area r = 0.910, *P* < 0.01; tricuspid valve circumference r = 0.905, *P* < 0.01(Fig. 4)). The mitral and tricuspid orifices could be showed as round, rectangular, and ellipse when opened. The real-time dynamic 4D image video facilitated a better visualization of motion of atrioventricular valves. This also assured a better understanding of the anatomy of the atrioventricular valves.

In total, 19 fetuses with cardiac valve malformations were included in this study, in which 4D volumes were all available. Eleven fetuses of complete atrioventricular septal defect (CAVSD), one fetus of partial atrioventricular septal defect (PAVSD), two fetuses of Ebstein’s anomaly, five fetuses of mitral or tricuspid valve stenosis or atresia. Portions of these cases were complicated with severe extra-cardiac malformations or chromosome abnormalities. All cases were confirmed by autopsy findings, postnatal echocardiograms or surgery findings. The 4D rendered images were successfully obtained in 89% (17/19) cases, in which the abnormal shape and motion of the cardiac valves were displayed when performing the RT 4D image cine loops.

## Discussion

CHD is the most common congenital anatomical malformation in fetuses and neonates and is the main cause of morbidity and mortality in perinatal period [13]. Valve malformation is one of the more common types. The atrioventricular valves, as the delicate complex anatomical combination, are composed of annulus, leaflets, chordae tendineae and papillary muscles, and have important hemodynamic significance. The normal physiological functions depend on the complete anatomical structure, fine coordination and cooperation of each part. Structural and functional abnormalities will cause atrioventricular level reflux even the secondary changes such as volume load. Correct prenatal diagnosis can effectively reduce morbidity and mortality. Further understanding of the morphological structure of the valves will help improve the detection rate of valve malformations during prenatal screening.

Currently, all fetuses undergoing routine obstetric examinations in our hospital are demanded for fetal cardiac structure. According to the AIUM guidelines, a detailed fetal echocardiography should be performed when it is suspicion of the congenital heart disease or high-risk factors, such as fetal extracardiac malformations, maternal diabetes or familial inherited disorders [14]. Fetal echocardiography is the best method for the diagnosis of fetal congenital heart malformations. The method of continuous transverse scanning has been introduced in routine screening programs to rule out most cardiac malformations [14]. For decades, 2D echocardiography has been widely used in clinical screening of various fetal heart diseases and accepted by most patients due to its advantages of real-time, non-invasive and high-resolution. Although 2D echocardiography could not show the complete morphology of the valves, experienced sonographers can still combine 2D echocardiography and color doppler flow imaging (CDFI) to detect most valve abnormalities by careful scanning. For example, thickened or dome-shaped valve suggests the possible existence of valve stenosis, which obtained further confirming by increased flow and distal dilatation. Usually, the valve echo is dense, no obvious valve movement observed, and the CDFI does not detect obvious blood flow signals, which may indicate valve atresia. However, in some cases, the low resolution and sensitivity of CDFI may not be able to display very small blood flow, and it is difficult to make an accurate diagnosis of valve stenosis or atresia. Most sonographers can rely on 2D echocardiography and CDFI to distinguish the complete or partial atrioventricular septal defects (CAVSD or PAVSD). When it needed to differentiate from simple atrial septal defect, displaying the details of anatomical morphologic and the number of leaflets is particularly important. The valve anatomy is complex, which requires more professional experience and theoretical knowledge. For inexperienced sonographers, it is often unable to make a correct diagnosis of minimal valve leaflet lesions. In addition, high-quality diagnostic information usually requires high-quality diagnostic planes, which are often difficult to achieve due to the frequent effects of fetal movement and unfavorable positions of the fetuses. We emphasize the need to further improve the prenatal screening methods for valve malformations and obtain more detailed anatomical information.

The development of volume imaging technology has brought great progress for prenatal diagnosis, providing a novel check method to obtain intuitive stereoscopic visual image to better determine fetal heart abnormalities, which is easier and more convenient. The advanced volume imaging technology is the method that uses a volume probe to collect the volume data of the fetal heart on the basis of 2D ultrasound, and then performs 4D reconstruction image of the interested area. Over the past 10 y, STIC has greatly promoted the development of fetal heart examination and has been proficiently used in the screening and diagnosis of fetal heart valve diseases. Which can obtain all 2D fetal heart images at any point in a single cardiac cycle, then arranged in time series and played in the form of movie playback by analyzing the information of spatial and temporal dimension. The cardiac cycle is identified without ECG gating in this manner. The 4D images may display as multiplanar cross-sectional or surface rendering. The examiner could then obtain a sufficient number of standard planes for a comprehensive diagnosis by navigating and reslicing within the volumes, of which the volumes contain all the region of interest and detailed anatomical information. Theoretically, it can support the acquisition of infinite planes within the volume data, although some planes are not necessary for diagnosis.

The adult hearts usually rely on the short-axis view to evaluate atrioventricular valves. In fetuses, the atrioventricular valves can also be visualized and measured directly or indirectly on the 4CV and the short-axis view. But, the accuracy of the diameter estimation on the 4CV is poor and the short-axis view of the fetuses is often more difficult to obtain. Under the volume mode, the operator only needs to appropriately adjust the size of ROI in the area of interest and rotate the 3 axes to easily obtain the 4D reconstructed images of the atrioventricular valves coronal view. It is a benefit. The coronal view of the atrioventricular valves is the best view for observing the atrioventricular valves of the fetuses, which usually cannot display on 2D echocardiography. The perspective parallel to the atrioventricular valves orifices can clearly show the anatomical shape of the valve, the number of the leaflets and is more suitable for the measurement of valve orifices. Which is more conducive to the evaluation of valve stenosis, atresia and minor lesions. Combining stereoscopic depth visual effect can more intuitively observe lesions than 2D echocardiography. Many reports have described the role of STIC in fetal heart valve examination. Hu G et al. used STIC to successfully visualize the valve structures of normal and abnormal fetuses, indicated that the lower valve display rate in the late pregnancy in 4D mode may be influenced by the sound shadow of fetal ribs [10]. Role et al. successfully measured the atrioventricular valves area on the atrioventricular valves coronal view with STIC for the first time, indicated that the atrioventricular valves area was increased with the growth of the gestational age. Which had provided clinical reference range of atrioventricular valves [15]. Adriaanse BM et al. confirmed the previous opinion of Rolo et al. and proposed that the rectangular shape was also a common shape of atrioventricular valves orifices [16]. The mechanical probe with STIC has the low acquisition speed, and the artifacts often occurred on the B and C planes due to the fetal movement and maternal respiration, which reduces the quality of image acquisition. This is very important, because the lower quality collected data will directly affect the quality of the reconstructed image and the diagnostic results. Currently, STIC uses post-processing mode for 4D volume rendering. The previous report has described this technique as an easy to learn technique that beginners can usually master in less than an hour [10]. However, this mode is usually performed after the patient’s examination and cannot provide real-time diagnostic information as 2D echocardiography.

In recent years, the emerging field of 4D echocardiography is the application of electronic matrix probes. The traditional mechanical probe is only composed of a row of crystals and the ultrasonic beam is deflected by a mechanical motor. Which the collection is slow, only suitable for low-speed movement in obstetric examination. For high-frequency fetal heart, although STIC has tried to be compatible with high-frequency fetal heartbeats and relatively low acquisition speed, it is still susceptible to artifacts during the acquisition process. The newly developed electronic matrix probe allows rapid examination of the fetal heart to obtain high-quality acquisition data. The probe is composed of multiple rows of crystal structures and deflects the ultrasonic beam by electronic scanning. The multi-row crystal structure has high-speed calculation abilities and can obtain four times faster acquisition speed than conventional mechanical probe [17]. Fast acquisition can suppress the influence of fetal movement and maternal respiration, improve the quality of volume data acquisition to obtain higher-quality reconstructed images. One of the most exciting technologies is the ability to acquire fetal heart volume data in real-time mode, enabling fetal heart examination into a real real-time field. Different from STIC technology, the RT 4D volume imaging is not an infinite loop of a single cardiac cycle, but the real-time four-dimensional images of the real cardiac cycle. When the traditional STIC collects volume data, pregnant women are usually asked to hold their breath and when there is no obvious fetal movement to choose volume data collection. Using the RT 4D mode, the operator has the ability to appropriately adjust the angle and direction of the probe to scan the area of interest and obtain high-quality volume data sets under the condition of fetal movement. It also has an ability to perform the analysis and processing in real-time to obtain real-time diagnosis information as 2D echocardiography and save the inspection time.

As far as we know, there is no study to evaluate fetal heart valve in prenatal examination using RT 4D volume imaging with electronic matrix probe. In our study, the RT 4D mode surface rendering is used to visualize the morphology of normal and abnormal atrioventricular valves, measure the circumference and area in normal fetuses, and evaluate the correlation with gestational age. Area and perimeter are both important indicators for size assessment. Previous some reports only noted the area and ignored the circumference [15, 16]. We propose that the combination of the two indicators can provide a more standard reference range for the better clinical diagnosis of stenosis and atresia valve malformations. It was also found that round, ellipses, and rectangles are common in the atrioventricular valves orifices during the study, which is consistent with the previous report of Adriaanse BM [16]. Combined with the tomographic imaging, chordae tendineae and papillary muscles can also be clearly shown. In addition, some atrioventricular valves structure abnormalities were successfully visualize using the real-time volume imaging with electronic matrix probe in our study.

**Fig. 5:**
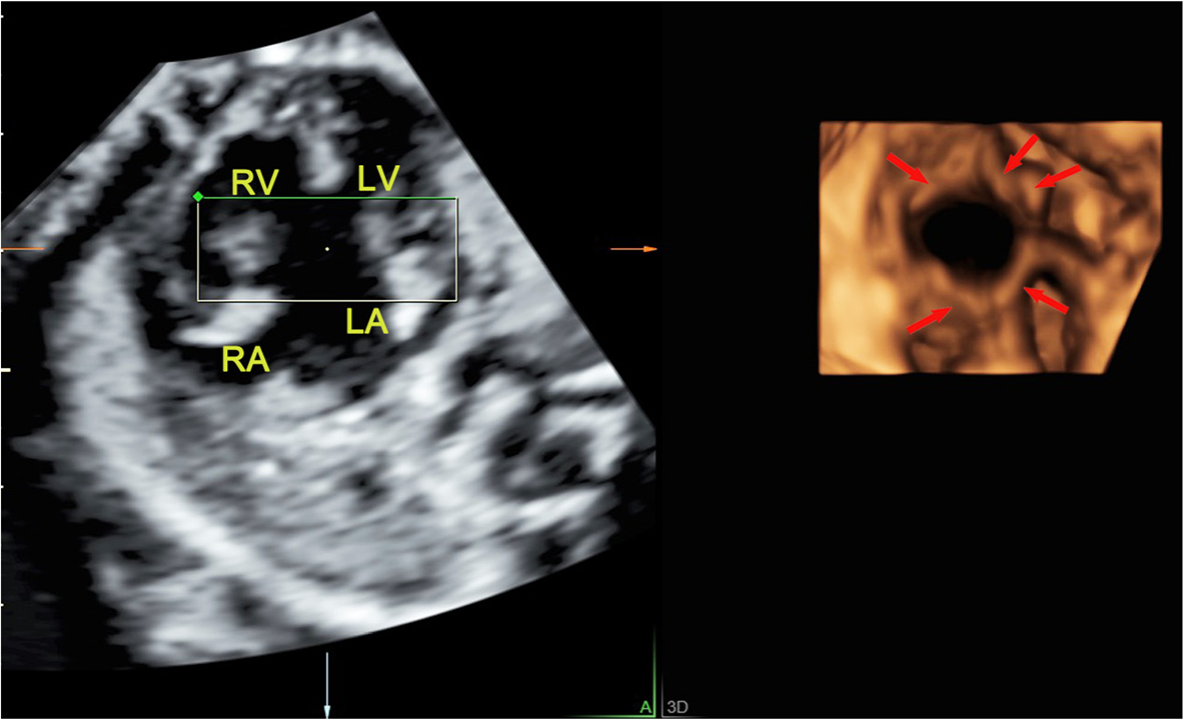
The 4D surface rendered image of atrioventricular valves obtained from reconstruction of RT 4D volume imaging in a fetus with complete atrioventricular septal defect. The 4D image can clearly show the common atrioventricular valves with five leaflets as a group of petal-like structures (indicated by arrows) during diastole. LA, left atrium; LV, left ventricle; RA, right atrium; RV, right ventricle

**Fig. 6:**
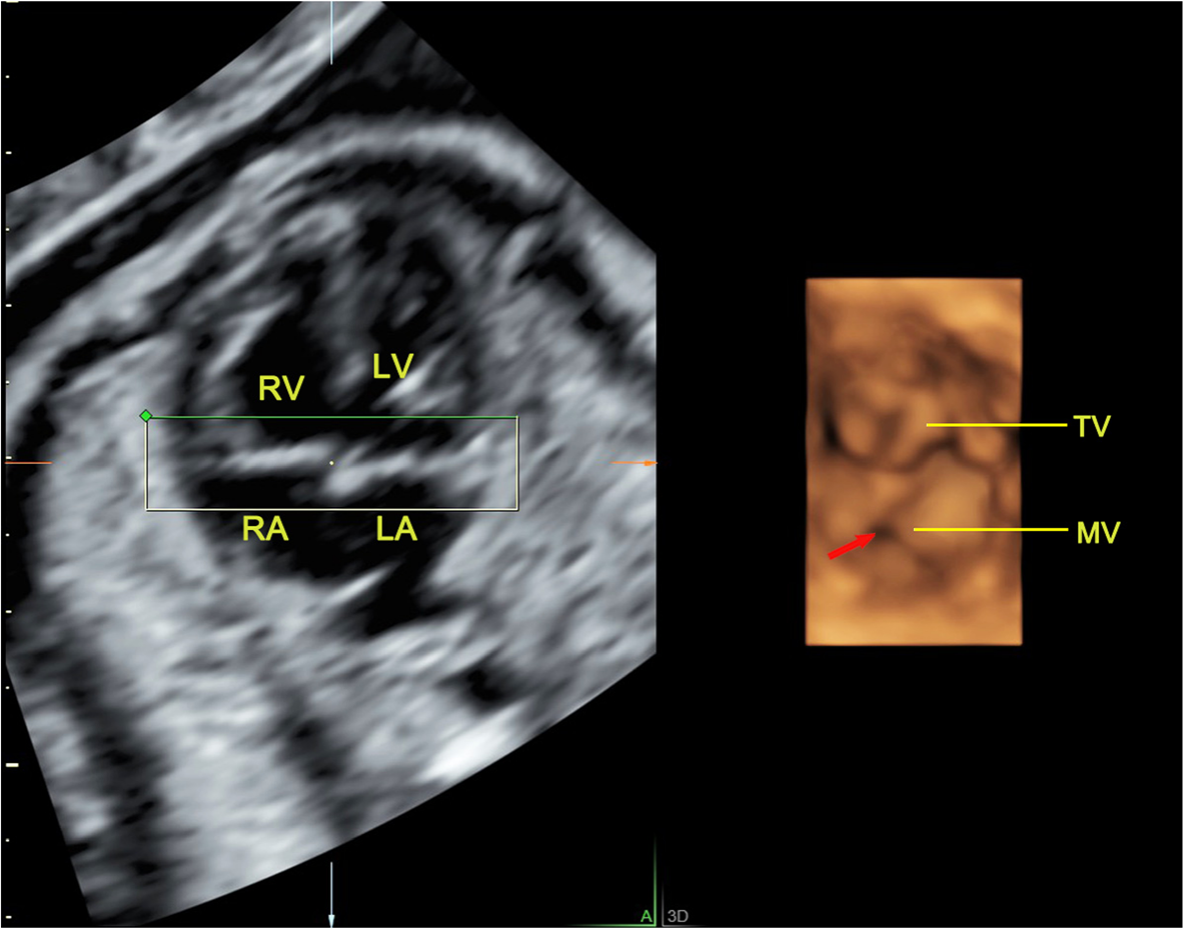
The 4D surface rendered image of atrioventricular valve obtained from reconstruction of RT 4D volume imaging in a fetus with partial atrioventricular septal defect. The 4D image can clearly show the two separate valve annuluses and two separate valves. The mitral valve with cleft (indicated by arrow). LA, left atrium; LV, left ventricle; MV, mitral valve; RA, right atrium; RV, right ventricle; TV, tricuspid valve

**Fig. 7:**
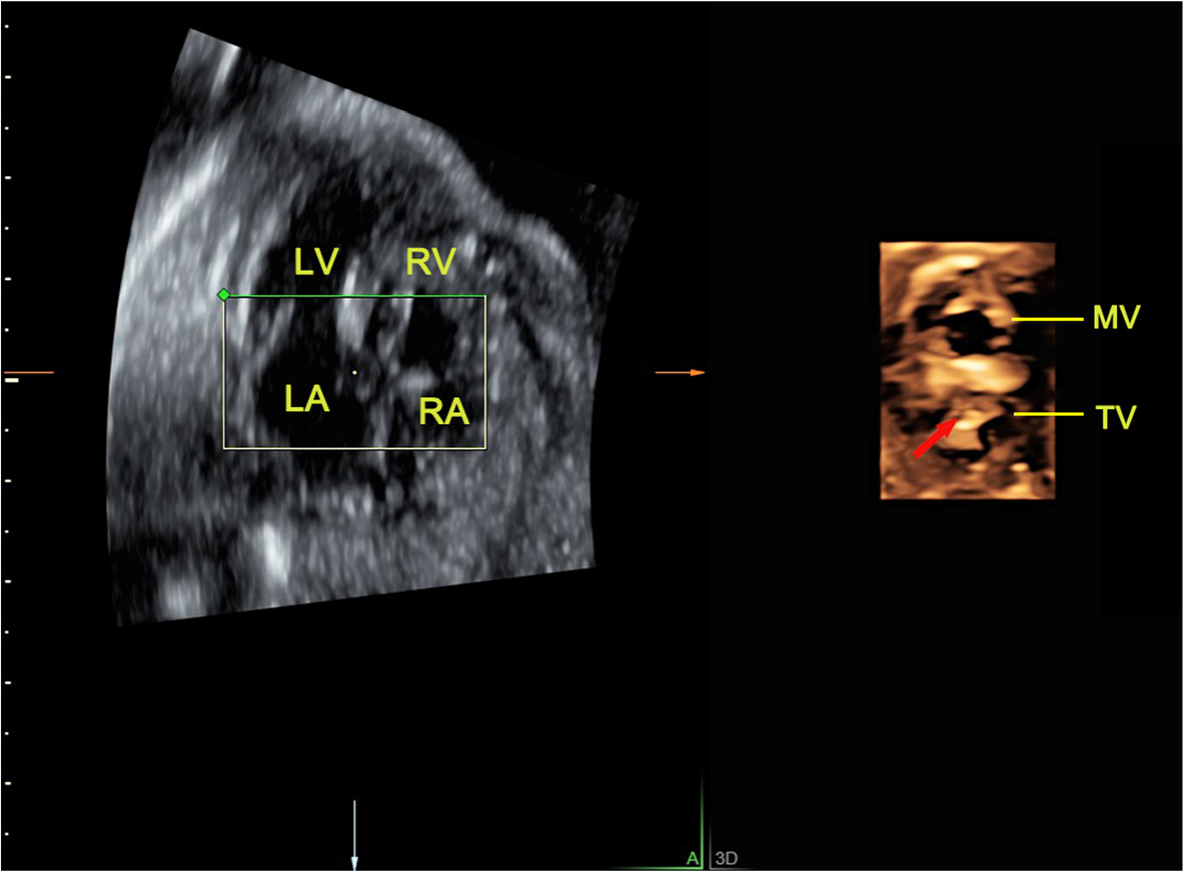
The 4D surface rendered images of atrioventricular valves obtained from reconstruction of RT 4D volume imaging in a fetus with tricuspid valve atresia. A thick echo dense membrane (indicated by arrow) could be visualized at the place of tricuspid valve orifice during diastole, while the shape of mitral valve was normal at the side. LA, left atrium; LV, left ventricle; MV, mitral valve; RA, right atrium; RV, right ventricle; TV, tricuspid valve

**Fig. 8:**
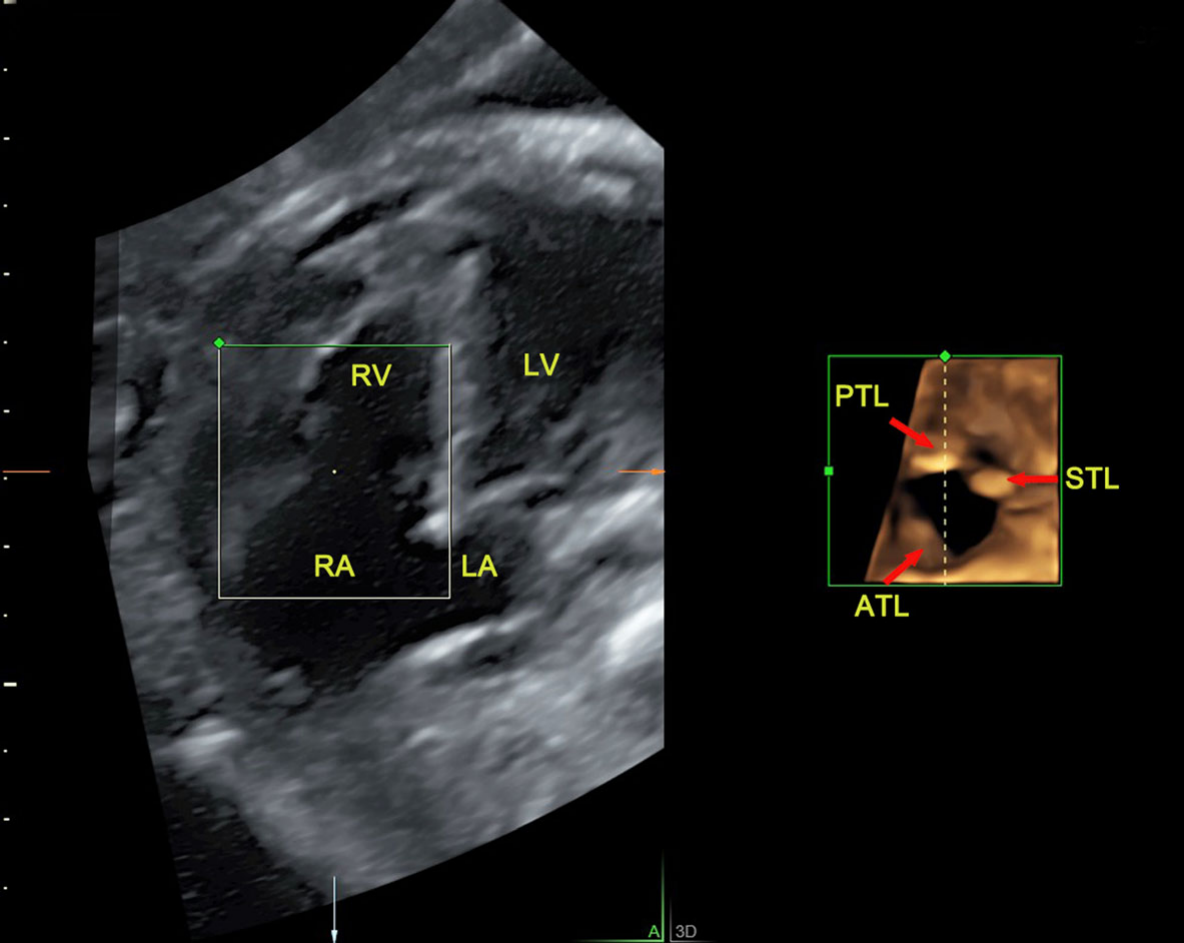
The 4D surface rendered image of tricuspid valve obtained from reconstruction of RT 4D volume imaging in a fetus with Ebstein’s anomaly. The view direction was set from the apex toward the bottom of the heart. The 4D rendered images showed the three leaflets of the tricuspid valve are not at the same annulus level. The anterior leaflet was longer while the septal and posterior leaflets were both shorter in size and moved from the tricuspid annulus down to the ventricular septum and right ventricular wall respectively (indicated by arrows). ATL, anterior tricuspid leaflet; LA, left atrium; LV, left ventricle; PTL, posterior tricuspid leaflet; RA, right atrium; RV, right ventricle; STL, septal tricuspid leaflet

There are many types of atrioventricular valves malformations, including CAVSD or PAVSD, mitral valve cleft, mitral and tricuspid valve stenosis or atresia and the downward displacement of tricuspid valve. Usually, CAVSD is easier to get a diagnosis. A single valve annulus together with a single valve, which can be identified from the RT 4D rendered images as a group of petal-like structures (Fig. 5). PAVSD has two separate valve annuluses and two separate valves, often accompanied with valve cleft which were all clearly visualized in the 4D reconstructed images (Fig. 6). By measuring valve orifice area and circumference in the end-diastolic, which are significantly less than the corresponding gestational age, it is easy to make a diagnosis of mitral or tricuspid valve stenosis. But it is often difficult to distinguish between serious stenosis or atresia of the mitral and tricuspid valves, which has similar manifestations usually accompanied with left or right ventricular dysplasia. In the 4D mode, the diagnosis of valve atresia can be easily made when a thick echo dense membrane structure is observed in the valve orifice, where no valve movement could be visualized, while the opening and closing of the other normal valve aside made a deep contrast (Fig. 7). Our study included the cases of downward displacement of the malformed tricuspid valve, also called Ebstein’s anomaly. The abnormality of attachment point of tricuspid valve is the main symptom and is usually seen in septum and posterior leaflets. The tricuspid valve septal leaflet can be clearly shown on the 4CV. However, the observation of the posterior leaflet of the tricuspid valve usually requires observation on the right ventricular inflow tract view, which is an unconventional view usually difficult for inexperienced sonographers. The serious downward displacement of the malformed tricuspid valve usually accompanies with the tricuspid valve dysplasia. Using RT 4D volume imaging, we can intuitively observe the abnormal attachment point of the septal and posterior leaflets. They move from the tricuspid annulus down to the ventricular septum and right ventricular wall respectively in one case (Fig. 8, Additional file 2).

In fact, RT 4D volume imaging has some advantages over 2D ultrasound. It may be easy for echocardiographers to display more anatomical details which could not be achieved by 2D ultrasound. RT 4D technology also has the better correlation with the surgery or autopsy findings than 2D ultrasound. The 4D rendered images are valuable in providing additional information during prenatal evaluation, such as the better consultations with obstetricians, cardiac surgeons with parents, especially for the surgeon in surgical planning. Currently, atrioventricular valves repairment is the critical step of AVSD surgical repair [18]. The mode of operation is determined by the exact morphology and anatomical details of the atrioventricular valves. The anatomy of the atrioventricular valves leaflets varies in size and number greatly. RT 4D technology enable to help the surgeon to identify the morphology and the size of the superior bridging leaflet directly. Usually, a small bridging leaflet with a large left mural leaflet has significant regurgitation. It is often needed for the reoperation after repair. In addition, the cleft can be easily identified by RT 4D mode than 2D ultrasound which is the key to distinguish PAVSD and simple atrial septal defect. PAVSD usually needed the further valve repairment. Ebstein’s anomaly can be treated with valvuloplasty. The prognosis mainly depends on the severity of the leaflets [19]. RT 4D technology can make a more complete evaluation of the tricuspid valve by displaying three leaflets simultaneously. It is more benefit that the abnormal attachment of the leaflet can be visually observed than 2D ultrasound, especially for the tricuspid posterior leaflet.

The limitation of RT 4D mode is that the image resolution is still lower than the 2D echocardiography although the frame rate has been significantly improved. It has been reported in the literature that some adjustments such as changing volume acquisition rate and resolution filters may improve the above problem [20]. How to obtain high-quality volume data is the key to imaging, and the quality of volume data is closely related to the quality of the 2D initial image. The electronic matrix probe 4D technology can only inhibit the influence of fetal movement but has no effect on the adverse position factors of the fetuses. Further advances in technologies in the future may overcome the limitations of current research.

## Conclusion

In summary, we proposed a novel method that allows the examiners to obtain high-quality 4D rendering images of the fetal cardiac valves in real-time efficiently and easily. Combined with the surface mode, the stereoscopic morphological structure of atrioventricular valves could be displayed in real-time, dynamically and multi-angle so as to obtain more detailed valve information for better screening and understanding of diseases. In addition, it was found that normal fetal atrioventricular valves area and circumference were positively correlated with gestational week, which could provide a standard reference range for clinical diagnosis.

## Supplementary Information

**Additional file 1:.** Video. The 4D cine loops obtained from RT 4D volume imaging with electronic matrix probe in a normal fetus. LA, left atrium; LV, left ventricle; MV, mitral valve; RA, right atrium; RV, right ventricle; TV, tricuspid valve.

**Additional file 2:.** Video. The 4D cine loops obtained from RT 4D volume imaging with electronic matrix probe in a fetus with Ebstein’s anomaly. ATL, anterior tricuspid leaflet; LA, left atrium; LV, left ventricle; PTL, posterior tricuspid leaflet; RA, right atrium; RV, right ventricle; STL, septal tricuspid leaflet.

## Data Availability

The datasets supporting the conclusions of this article are included within the manuscript (and its additional files). The authors would like to share raw anonymized video data related to the current study, which could only be used for personal study. The demanders may contact baogoubei@hotmail.com.
